# The HDAC interaction network

**DOI:** 10.1038/msb.2013.33

**Published:** 2013-06-11

**Authors:** Ilana Livyatan, Eran Meshorer

**Affiliations:** 1 Department of Genetics, The Alexander Silberman Institute of Life Sciences, The Hebrew University of Jerusalem, Edmond J. Safra Campus, Jerusalem, Israel

The new age of social networking has taught us that a lot can be inferred about you and me just based on our ‘friendship network'. Biochemists have been exploiting this approach for years when trying to discover the functional roles of proteins. Protein–protein interactions are the basis for the diverse cellular functionality that builds a living organism, manifested in multi-protein complexes, signal–transduction pathways and other protein effector pathways. As protein function heavily relies on these interactions, in many cases the function of a protein can be inferred from the list of its interacting partners. In a recent article published in *Molecular Systems Biology*, Joshi et al (2013) describe a novel systematic approach integrating advanced proteomic and computational methods for uncovering specific protein interactions. Applying this new methodology, they built a comprehensive network of functional protein interactions for 11 members of the histone deacetylase (HDAC) protein family, filling an important gap in our basic understanding of how HDACs affect cellular processes, are involved in disease, and can be targeted with drugs to alleviate disease phenotypes.

HDACs are a family of enzymes responsible primarily for deacetylating lysine residues within histones, although they often act on non-histone proteins as well, and many are not confined to the cell nucleus. HDACs are divided into five groups based on their sequence similarity and function. Class I is comprised of HDAC1, 2, 3 and 8, all of which have the simplest structure; class IIa includes HDAC4, 5, 7 and 9, which have large N-terminal extensions; class IIb consists of HDAC6 and 10, with extended C-termini; class III comprises of the Sirtuins (SIRT1–7), and class IV includes a single member, HDAC11, which shares features with both class I and class II HDAC enzymes (Haberland et al, 2009). When they operate on histone proteins, which package nuclear DNA into chromatin, the DNA generally wraps more tightly around the histone proteins once the acetyl group is removed, making some DNA regions more accessible (‘euchromatin' or ‘active chromatin') and some less accessible (‘heterochromatin' or ‘suppressive chromatin') to other proteins and the transcriptional machinery. This simple chemical modification has considerable effects as DNA accessibility is a major regulator of all DNA-related mechanisms, particularly gene expression. Acetylated lysines are recognized by a set of proteins, containing either a bromodomain or a tandem PhD-finger domain, and which can recruit additional protein complexes that modify the expression pattern of the specific region. As the transcriptional program of the cell largely defines its identity, any changes in DNA expression patterns can result in something as remarkable as embryonic development or as devastating as out-of-control metastatic cancer. Therefore, it is not surprising that perturbations of HDAC functions have profound effects on a variety of cellular processes, including cell cycle regulation (Telles and Seto, 2012), stem cell differentiation (Hezroni et al, 2011), development (Reichert et al, 2012), and memory and brain function (Guan et al, 2009; Sailaja et al, 2012). Consequently, HDACs have turned into promising targets for the treatment of multiple diseases including, but not limited to, neurodegenerative diseases (e.g., Huntington's disease (McFarland et al, 2012) and other PolyQ diseases (Cohen-Carmon and Meshorer, 2012)) and cancer (Barneda-Zahonero and Parra, 2012), while small-molecule inhibitors of HDACs are being increasingly used in research of such diseases. Several HDAC inhibitors including SAHA (vorinostat) and valproic acid have even been FDA approved for advanced cutaneous T-cell lymphoma, and for neurological symptoms such as epilepsy, respectively.

Despite the exponential growth in the number of publications related to HDACs, the question of whether their main role is to regulate gene expression remains open. How do these enzymes, especially the ones that are closely related in sequence and structure, achieve specificity? What controls their nuclear versus cytoplasmic localization?

To start answering some of these questions Joshi et al (2013) used a novel proteomic approach to identify the protein interaction networks of HDACs in T cells. Specific protein interactions were determined by a label-free affinity purification followed by mass spectrometry-based proteomics approach followed by downstream computational analysis using a modeling algorithm. A complementary metabolic labeling strategy, based on the SILAC (stable isotope labeling by amino acids) technology, was used to ascertain the stability of the identified interactions. This integrative hybrid approach led to the identification of over 200 novel protein interactions with the HDAC family, thus linking HDACs to a variety of functional processes beyond chromatin and epigenetic gene regulation (Figure 1). These include RNA processing, protein ubiquitination, signal transduction and nuclear import, just to name a few. Independent analysis of the HDAC11 protein network revealed association with the SMN protein subnetwork that affects RNA splicing of minor introns, suggesting that, when misregulated, the HDAC11 network can contribute to the phenotype of spinal muscular atrophy patients. Downregulation of HDAC11 recapitulated splicing defects associated with the downregulation of the SMN1 member of the SMN complex, providing a new avenue of association between HDACs and disease.

The HDAC network published in this study provides a sorely needed platform for research on HDAC functions outside of the DNA expression box. Going forward, research efforts can now investigate the specific roles of individual HDACs in a broad variety of cellular processes and uncover the respective underlying molecular mechanisms. Further work is required in order to distinguish the proteins that work in complex with HDACs from those that are substrate targets. Additionally, establishing the HDAC interaction network outside of T cells will facilitate the analysis of its function beyond T-cell biology. One of the most important future outcomes should be the ability to propose specific targets for inhibition of specific mechanisms, thus allowing safer and more targeted pharmaceutical approaches to diseases involving HDACs as well as facilitating advanced research of biological processes.

## Figures and Tables

**Figure 1:**
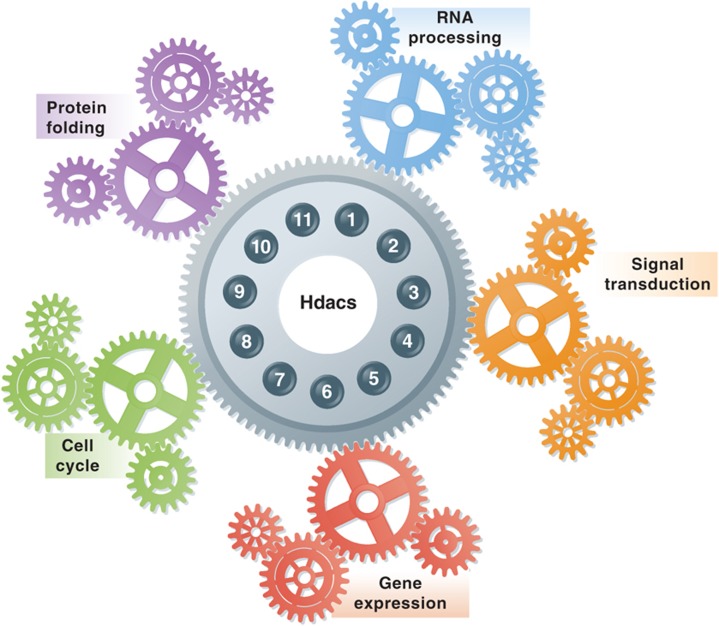
The newly identified interactome of the HDAC family links HDACs to a variety of functional processes beyond chromatin and epigenetic gene regulation.
